# Mechanisms for Precision, Patient-Centered Therapy in Inflammatory Bowel Disease

**DOI:** 10.3390/biomedicines13102504

**Published:** 2025-10-14

**Authors:** Dingpei Long

**Affiliations:** 1 State Key Laboratory of Resource Insects, Institute of Sericulture and Systems Biology, Southwest University, Chongqing 400715, China; dplong@swu.edu.cn; 2 Digestive Disease Research Group, Institute for Biomedical Sciences, Georgia State University, Atlanta, GA 30303, USA

## 1. Introduction

Inflammatory bowel disease (IBD), encompassing Crohn’s disease (CD) and ulcerative colitis (UC), arises from genetic susceptibility, environmental triggers, dysbiotic microbiota, and mucosal immune dysregulation [1,2,3,4]. Aberrant innate and adaptive immune responses to commensals drive chronic injury, while bile acid signaling and epithelial barrier failure further modulate inflammation [5,6,7]. Dysbiosis extends across gut and oral niches. Mucosa-associated microbial shifts are linked to drug tolerance, and the detection of oral fungi and herpesviruses in IBD suggests a potential oral–gut axis [8,9,10,11]. Epidemiology shows a rising global burden with substantial complications and cancer risk, reinforcing treat-to-target care and multidisciplinary management of extraintestinal manifestations (EIMs) [3,12,13].

Diagnostic approaches are shifting from invasive endoscopy toward noninvasive biomarkers and histology: CRP and fecal calprotectin are widely used to monitor disease activity, while histologic healing correlates with outcomes and is increasingly pursued as a therapeutic goal [13,14,15]. Therapeutics have expanded beyond 5-ASA, steroids, and thiopurines to include biologics (anti-TNF, anti-integrin, anti-IL-12/23) and oral small molecules (JAK, S1P modulators), thereby improving remission rates and promoting mucosal healing. However, many patients still experience primary nonresponse, loss of response, or adverse events [7,8,16,17]. Real-world considerations include the possibility that co-therapy potentially attenuating vedolizumab effectiveness, as well as the use of pediatric combination strategies (e.g., methotrexate with infliximab) [18]. Lifestyle and dietary guidance merit individualized approaches; for instance, coffee is often neutral or beneficial rather than uniformly contraindicated [19]. Precision delivery and chemoprevention concepts are advancing via oral nano- and microtechnologies and biomaterials (including silk-based systems), with preclinical efficacy for colitis and colitis-associated neoplasia [20,21,22]. Moreover, comorbidities such as irritable bowel syndrome (IBS) overlap, fibromyalgia/chronic fatigue, and obesity with its proinflammatory effects further shape outcomes and care pathways [23]. Altogether, this Special Issue (Second Edition) comprises 18 articles that collectively traverse pathophysiology, diagnostics, and therapy, bridging laboratory insights with pragmatic clinical decision-making.

## 2. Cross-Cutting Themes: Toward Precise, Mechanism-Guided Care

**Mechanistic convergence and tractable targets**. A representative preclinical study demonstrates that inhibiting caspase-1-dependent pyroptosis via TLR4/NF-κB and inflammasome pathways attenuates colitis and repairs barrier function, positioning pyroptosis as a promising therapeutic axis in UC [24]. Complementing innate immune targets, a narrative synthesis highlights epithelial, immune, and microbial crosstalk as the foundation for both current and emerging therapies [25], while a state-of-the-art review details how bile acids operate as signaling metabolites with therapeutic leverage points across receptors and metabolic circuits [26].

**Microbiome-aware management**. Multiple studies illustrate that mucosa-associated dysbiosis and even extra-intestinal microbial niches may shape clinical phenotypes and treatment tolerance. A study in UC links 5-aminosalicylic acid intolerance with specific mucosal microbial signatures [27], whereas a study in CD describes how commonly used therapies (5-ASA, azathioprine, infliximab) are associated with shifts toward short-chain-fatty-acid producers [28]. Extending beyond the gut, a focused study catalogs emerging oral fungi and herpesviruses in IBD, underscoring an oral–gut axis relevant to opportunistic infection risk [29]. At the same time, a real-world analysis of dietary habits finds that coffee appears to be neutral or even beneficial for inflammation in many patients, supporting individualized counseling over blanket restriction [30]. Finally, a forward-looking review **distills microbiome targets and strategies** under development, from refined FMT to next-generation probiotics [31].

**Model-informed therapeutics and precision selection**. Pharmacologic contributions emphasize not only ***which*** drug to use, but ***how*** to use it. Physiologically based pharmacokinetic (PBPK) simulations raise route- and weight-specific considerations for infliximab, cautioning that flat subcutaneous dosing may under-expose heavier adults, while weight-band dosing is more appropriate in pediatric settings [32]. A formal external evaluation of vedolizumab population PK models underscores both current limitations and their utility for simulation-based dose planning [33]. A meta-analysis clarifies that subcutaneous and intravenous IL-12/23 blockades perform comparably for induction and maintenance in moderate-to-severe CD, enabling preference- and resource-sensitive route choice [34]. Beyond PK, whole-exome sequencing identifies variants enriched among vedolizumab non-responders (e.g., in classical IBD-immunity genes), foreshadowing pharmacogenetic triage for biologics [35]. A newly added genetics study identifies shared etiologies between IBD and several other immune-mediated diseases and reveals novel loci with functional annotations and mechanistic plausibility; these finding reinforce the importance of genotype-informed therapeutic stratification [36].

**Patient-centered outcomes and systemic disease**. Several reviews remind us that IBD is a systemic disorder: extraintestinal manifestations (EIMs) require multidisciplinary strategies [12], with ocular complications demanding coordinated gastro-ophthalmology care and awareness of therapy-related effects [37]. A population-based structural equation analysis shows that perceived health → control → acceptance is a reproducible pathway; in high-burden IMIDs such as IBD, acceptance exerts a particularly strong influence on lived experience, supporting the integration of psychosocial support into routine care [38].

## 3. Overview of the 18 Articles by Domain

### 3.1. Mechanisms and Preclinical Therapeutics

- **Pyroptosis inhibition in UC (original research)**. Inhibiting caspase-1-dependent pyroptosis through TLR4/NF-κB and inflammasome modulation reduces colitis and restores barrier integrity, highlighting pyroptosis as a promising druggable target [24].
- **Pathogenesis synthesis (review)**. This narrative review integrates epithelial, immune, microbial, and environmental cues to map IBD pathobiology and explores the resulting therapeutic implications [25].
- **Bile-acid signaling (review)**. Bile acids are reframed as signaling mediators with entry points for intervention at both the receptor and metabolic levels [26].

### 3.2. Microbiome, Diet, and Mucosal Healing

- **Mucosa-associated microbiota and drug intolerance (original research).** Patients with UC who have 5-ASA intolerance exhibit distinct mucosal microbiota compositions, suggesting that first-line treatment choices could be stratified based on microbiome profiles [27].
- **Drug–microbiome interplay in CD (original research).** Mesalazine, azathioprine, and infliximab use is associated with shifts toward short-chain fatty acid (SCFA)-producing taxa and greater microbial evenness relative to untreated disease [28].
- **Oral pathobionts in IBD (original research).** Fungal and viral colonization of the oral cavity is documented in IBD, highlighting a potential extraintestinal reservoir of dysbiosis [29].
- **Dietary perspective: coffee (original research).** In a real-world cohort, many patients continue coffee consumption and do not experience worsened symptoms. In UC, coffee users were observed to have lower fecal calprotectin levels, suggesting a benign or even beneficial effect [30].
- **Microbiome-targeted therapies (review).** This review provides an overview of fecal microbiota transplantation (FMT) refinement, live biotherapeutics, and engineered strategies aligned to host–microbe mechanisms [31].

### 3.3. Clinical Pharmacology and Comparative Effectiveness

- **Infliximab PBPK (original research).** Virtual adult and pediatric populations show route- and weight-dependent exposure differences; notably, flat subcutaneous (SC) dosing may underdose heavier adults, advocating for exposure-guided adjustments [32].
- **Vedolizumab population-PK models (original research).** An external evaluation suggests that existing models are imperfect for a priori individualization but are useful for simulation-guided regimen planning and, with therapeutic drug monitoring (TDM), for dose optimization [33].
- **IL-12/23 inhibitors: SC vs. IV (systematic review/meta-analysis).** Comparable efficacy and safety across routes support delivery aligned with patient preference in moderate-to-severe CD [34].
- **Pharmacogenetics of vedolizumab response (original research).** Whole-exome sequencing (WES) in a Middle Eastern cohort identifies rare variants enriched in non-responders across canonical IBD-inflammation genes, pointing to biomarker-guided biologic selection [35].

### 3.4. Clinical Management and Complications

- **Postoperative recurrence in CD (review/meta-analysis).** Risk stratification, endoscopic monitoring, and early biologic prophylaxis—often using an anti-TNF agent as first-line therapy—serve as the cornerstones of recurrence prevention and management [39].
- **Extraintestinal disease (review).** A comprehensive review of EIMs (extraintestinal manifestations) outlines the pathophysiology and treatment principles across joints, skin, eyes, and the hepatobiliary system, emphasizing the need for integrated care [12].
- **Ocular involvement (review).** Episcleritis tends to track with luminal disease activity, whereas uveitis often diverges. Notably, therapy can both treat ocular complications and, in rare cases, precipitate them—care pathways should anticipate both possibilities [37].

### 3.5. Genetics and Biomarkers

- **Shared genetic etiologies across IMIDs (original research—newly added).** Multi-trait genome-wide association studies (GWAS) and integrative analyses reveal significant genetic relationships between IBD and several other immune-mediated inflammatory diseases (e.g., axial spondyloarthritis, psoriasis, uveitis/iridocyclitis, psoriatic arthritis). These analyses also identify new loci with functional annotations. Together, these findings underscore a germline foundation for precision stratification [36].
- **Diagnostic/functional candidates in UC (original research).** SLC26A2 is downregulated in active UC and is linked to IL-17 signaling and epithelial barrier pathways, suggesting significant diagnostic and therapeutic relevance [40].

## 4. Looking Ahead

Priorities converge on mechanism-guided, model-informed, patient-centered precision care. First, mechanistic hypotheses (e.g., pyroptosis, epithelial transport, bile acid circuits) should be embedded in clinical trials and combination therapeutic strategies to move beyond the ceiling of monotherapy [6,7,41]. Second, precision dosing and route selection should be operationalized using PBPK and population-PK tools that support exposure-guided use of infliximab and vedolizumab. Subcutaneous vs. intravenous IL-12/23 blockade shows comparable efficacy, thereby enabling delivery based on patient preference [7,14,42,43]. Furthermore, pharmacogenetics data (for example, WES signals linked to vedolizumab nonresponse) should inform triage and sequencing. Third, treatment targets must broaden to include histologic remission, biomarker normalization, dysbiosis correction, and improved quality of life, aligning with treat-to-target frameworks and concurrent EIM management [12,13,14,15,43].

Microbiome-directed care is progressing from proof-of-concept to standardized platforms: multidonor FMT, defined consortia, and probiotic- and metabolite-based strategies tailored to host–microbe states [2,9,19]. Regenerative and interventional solutions—from mesenchymal cell therapies for fistulizing disease to advanced surgical-biologic hybrids—should be integrated with precision medicine pathways [41,43,44]. Real-world modifiers (e.g., concomitant proton pump inhibitor use), pediatric-to-adult transitions, and the immunometabolic impacts of obesity all warrant proactive management [18,45]. Adjacent innovations, oral nanomedicine and biomaterials for targeted delivery and chemoprevention, can reduce systemic exposure while sustaining mucosal control [20,21,22]. Finally, combination and sequencing algorithms—supported by decision frameworks and shared decision-making—aim to deliver durable, steroid-free remission with minimal complications for patients across diverse IBD phenotypes [13,17,41,43].
